# Protective Effects of *Abrus cantoniensis* Hance on the Fatty Liver Hemorrhagic Syndrome in Laying Hens Based on Liver Metabolomics and Gut Microbiota

**DOI:** 10.3389/fvets.2022.862006

**Published:** 2022-04-15

**Authors:** Xu Liu, Yinchuan Pan, Youming Shen, Hailong Liu, Xinghua Zhao, Jianyong Li, Ning Ma

**Affiliations:** ^1^College of Veterinary Medicine, Veterinary Biological Technology Innovation Center of Hebei Province, Hebei Agricultural University, Baoding, China; ^2^Research Institute of Pomology, Chinese Academy of Agricultural Sciences, Xingcheng, China; ^3^Institute of Animal Husbandry and Veterinary Medicine, Hainan Academy of Agricultural Sciences, Haikou, China; ^4^Key Lab of New Animal Drug Project of Gansu Province, Key Lab of Veterinary Pharmaceutical Development of Ministry of Agriculture and Rural Affairs, Lanzhou Institute of Husbandry and Pharmaceutical Science of Chinese Academy of Agricultural Sciences, Lanzhou, China

**Keywords:** lipid metabolism, high-energy low-protein diet, phytochemicals, metabolic profiling, herbal medicine

## Abstract

As a metabolic disease, fatty liver hemorrhagic syndrome (FLHS) has become a serious concern in laying hens worldwide. *Abrus cantoniensis* Hance (AC) is a commonly used plant in traditional medicine for liver disease treatment. Nevertheless, the effect and mechanism of the decoction of AC (ACD) on FLHS remain unclear. In this study, ultra-high performance liquid chromatography analysis was used to identify the main phytochemicals in ACD. FLHS model of laying hens was induced by a high-energy low-protein (HELP) diet, and ACD (0.5, 1, 2 g ACD/hen per day) was given to the hens in drinking water at the same time for 48 days. Biochemical blood indicators and histopathological analysis of the liver were detected and observed to evaluate the therapeutic effect of ACD. Moreover, the effects of ACD on liver metabolomics and gut microbiota in laying hens with FLHS were investigated. The results showed that four phytochemicals, including abrine, hypaphorine, vicenin-2, and schaftoside, were identified in ACD. ACD treatment ameliorated biochemical blood indicators in laying hens with FLHS by decreasing aspartate aminotransferase, alanine aminotransferase, triglycerides, low-density lipoprotein cholesterol, and total cholesterol, and increasing high-density lipoprotein cholesterol. In addition, lipid accumulation in the liver and pathological damages were relieved in ACD treatment groups. Moreover, distinct changes in liver metabolic profile after ACD treatment were observed, 17 endogenous liver metabolites mainly associated with the metabolism of arachidonic acid, histidine, tyrosine, and tryptophan were reversed by ACD. Gut microbiota analysis revealed that ACD treatment significantly increased bacterial richness (Chao 1, *P* < 0.05; Ace, *P* < 0.01), and upregulated the relative abundance of *Bacteroidetes* and downregulated *Proteobacteria*, improving the negative effects caused by HELP diet in laying hens. Taken together, ACD had a protective effect on FLHS by regulating blood lipids, reducing liver lipid accumulation, and improving the dysbiosis of liver metabolomics and gut microbiota.

## Introduction

With high quality and low price, the egg is widely consumed in our daily life. However, fatty liver hemorrhagic syndrome (FLHS), which frequently occurred in laying hens, is seriously hampering the development of the poultry industry. As a metabolic disease, FLHS inhibits the synthesis and transport of low-density lipoprotein and the excretion of fatty acids from the liver, which results in lipid metabolism disorder and excessive lipid deposition in the liver (1). Many factors such as genetic factors, nutrition disorders, toxic substances, and feeding management are associated with the occurrence of FLHS (2, 3). Of note, FLHS has a negative effect on production performance and health in laying hens, especially for the caged laying hens with good body condition. FLHS can lead to a sudden drop in egg production and a shortened egg production peak (4). Moreover, a large number of previous research findings have indicated that FLHS is the main cause of the non-infectious mortality for caged layers (5, 6). Directly or indirectly, FLHS causes huge economic losses for farmers and the poultry industry. Therefore, it is imperative to find an effective treatment for reducing the damage of FLHS.

Medicinal herbs have been well-known for disease treatment and are important natural sources for novel bioactive compounds. As an edible vegetable and medicinal plant, *Abrus cantoniensis* Hance (AC) is a woody climbing shrub found in the southern regions of China, such as Guangdong and Guangxi provinces (7). AC contains lots of active ingredients such as flavonoids, alkaloids, terpenoids, and saponins. Abrine and hypaphorine, two alkaloids in AC, have been reported with a variety of pharmacological activities such as antioxidation, anti-inflammation, and anti-proliferation (8–10). Wang et al. found that the flavonoids in AC such as vicenin-2 and schaftoside showed antioxidant, anti-inflammatory, and lipid regulation properties (11). Moreover, it has been claimed that the AC has hepatoprotective activities, which can effectively clean liver toxicants and prevent hepatitis and other chronic liver diseases (12). However, up to data, there is no report about the efficacy and application of AC on FLHS in laying hens.

Through the identification and quantification of small molecules, metabolomics is a promising approach to explore the metabolic profile in biological systems. Nowadays, metabolomics has been widely applied in the studies of disease pathogenesis (13). Through metabonomic and pathway analysis, Zhuang et al. found that glucose, lipid, and amino acid metabolisms were disordered in the progression of FLHS induced by a high-energy low-protein (HELP) diet (14). Gut microbiota, the microbial communities inhabiting the gastrointestinal tract, plays an essential role in the immune function, physiology, and nutrition metabolism of the host. It has been proved that the dysbiosis of microbial community structure in the cecum is related to the altered metabolites, liver function, and severity of steatohepatitis in laying hens (15). Therefore, the regulation of metabolomic profile and gut microbiota is beneficial for the treatment of FLHS. It has been recognized that the combination of metabolomics and gut microbiota opens a new window to investigate the effects and mechanisms of drugs, especially for traditional Chinese medicine (16, 17). However, so far, none of the studies have applied metabolomics and gut microbiota to investigate the action mechanism of AC on FLHS.

In this study, the decoction of AC (ACD) was prepared, and its main ingredients were characterized by ultra-high performance liquid chromatography (UPLC) analysis. Subsequently, we investigated the pharmacological activity of ACD in laying hens with FLHS induced by the HELP diet through blood biochemical and histopathological analysis. In addition, integration of metabolomics and gut microbiota was employed to elucidate the underlying molecular mechanism of the beneficial effects of ACD. The results of this study can provide the basis for the application of AC in the treatment of FLHS.

## Materials and Methods

### Preparation and Characterization of Decoction of *Abrus cantoniensis* Hance

The AC was collected from Guangxi Province, China, authenticated by researcher Hailong Liu (Hainan Academy of Agricultural Sciences), and deposited in the veterinary medicine lab of Hebei Agricultural University with voucher specimen number (HEBAU-20201105). The AC was dried, cleaned, and then cut into 2 cm long pieces. The AC pieces were soaked for 1 h in 10 × (v/w) distilled water and then boiled for 2 h and filtered. Distilled water was mixed five times (v/w) to the residue and boiled for another 1 h and also filtered. The two filtrates were collected and merged, and then concentrated to 3 g/ml (crude drugs) to prepare ACD. The ACD was stored at 4°C for experimental use.

An Acquity UPLC H-CLASS (Waters Corporation, MA, USA) was used to further characterize the main components of ACD. Chromatographic separation of ACD was performed on the HSS T3 column (2.1 mm × 100 mm, 1.8 μm, Waters Corporation, MA, USA). The mobile phase was composed of A (water with 0.1% formic acid) and B (acetonitrile) with a linear gradient elution: 0–9 min, 98–90% A; 9–11 min, 90% A; 11–12 min, 90–86% A; 12–15 min, 86–85% A; 15–17 min, 85% A; 17–18 min, 85–83% A; 18–19 min, 83% A; 19–20 min, 83–98% A. The column temperature was maintained at 35°C and with a flow rate of 0.3 ml/min. The detection wavelengths were set at 270 nm, and the injection volume was set at 10 μl. Peaks were identified through the comparison of the retention time with the reference compound.

### Animal Treatment Procedure and Sample Collection

In this study, forty Jing Fen laying hens aged 90 days were used. After 2 weeks for acclimatization, the laying hens were randomly divided into five groups (8 animals per treatment), namely, control group (basal diet), model group (HELP diet), ACD low dose (ACD-L, HELP diet with 0.5 g ACD/hen per day), ACD medium dose (ACD-M, HELP diet with 1 g ACD/hen per day), and ACD high dose groups (ACD-H, HELP diet with 2 g ACD/hen per day). The ACD was administrated in drinking water for 48 consecutive days. Laying hens were subjected to 1 h water deprivation before administration, which could guarantee the ACD in water was completely taken by laying hens. The FLHS in laying hens was induced by the HELP diet as reported in previous studies (14, 18), and the detailed composition of the basal diet and HELP diet is shown in Table 1.

**Table 1:** Composition of the basal diet and HELP diet.

**Composition**	**Basal diet**	**HELP diet**
**(air-dry basis) %**		
Corn	64.00	70.00
Wheat bran	2.00	1.20
Soybean meal	24.00	14.58
Fat-soybean oil	0.00	4.22
Calcium	8.00	8.00
Premix^*^	2.00	2.00
Total	100.00	100.00

*HELP diet: high-energy low-protein diet*;

* *The composition of premix: multiple vitamins, 30 mg; zeolite powder, 6 mg; anti-oxidizing quinolone, 50 mg; methionine, 100 mg; choline, 90 mg; bacitracin zinc, 26.7 mg; bran, 350 mg; CuSO_4_, 4.6 mg; FeSO_4_, 28.4 mg; MnSO_4_, 35.46 mg; ZnSO_4_, 76 mg; Na_2_SO_3_, 5 mg; The basal diet was formulated according to the National Research Council (1994)*.

At the end of the experiment, the hens were fasted for 12 h and anesthetized with intravenous injection of sodium pentobarbital (50 mg/kg). Blood samples were collected from the brachial vein into vacuum tubes. The serum was obtained by centrifugation at 4°C of 4,000 g for 10 min and then stored at −20°C for further analysis. The liver and cecal contents of laying hens were carefully removed, collected, and snap-frozen in liquid nitrogen, and stored at −80°C for metabolomics and gut microbiota analysis, respectively. Meanwhile, a part of liver tissue was fixed in 4% formalin for the observations of pathological changes.

### Blood Biochemistry and Histopathological Analysis

Blood biochemistry indexes in serum such as triglycerides (TG), total cholesterol (TCH), high-density lipoprotein cholesterol (HDL-C), low-density lipoprotein cholesterol (LDL-C), alanine aminotransferase (ALT), and aspartate aminotransferase (AST) were analyzed by the commercial kits provided by Shanghai Jiang Lai bio-technology Co., Ltd (Shanghai, China). Under the standard protocol, the liver tissue was made and stained with HE, and oil red O to observe the pathologic changes. The pathological slides were examined using a 13395H2X microscope (Leica Co., Ltd, Wetzlar, Germany).

### Metabolomics Data Acquisition

Before metabolomics analysis, the liver tissues were thawed at room temperature. Next, 80 mg liver tissue were placed in polyethylene tubes, and 1 ml of cold methanol/acetonitrile/H_2_O (2:2:1, v/v/v) was added, and the mixture was adequately vortexed and homogenized for 1 min. The homogenate was ultrasonically extracted (30 min/once, twice, 4°C), incubated (1 h at −20°C), and then centrifuged (14,000 *g*, 20 min, 4°C). The supernatants were collected and then dried by nitrogen to obtain a lyophilized powder. For instrumental analysis, lyophilized samples were reconstituted by dissolving in 100 μl acetonitrile/water (1:1, v/v), and an aliquot of 2 μl was injected for further analysis. To monitor the stability of the system and evaluate the reliability of experimental data, three quality control (QC) samples were prepared by pooling 10 μl of each liver sample and analyzed with the same procedure.

Metabolomics analysis was performed with an Agilent 1290 UPLC (Agilent Technologies Inc., CA, USA) coupled with Triple TOF 6600 system (AB SCIEX, Foster City, USA). Mass-grade acetonitrile, methanol, and formic acid were purchased from Merck (Darmstadt, Germany). Deionized water (18 MΩ) was prepared with a Direct-Q®3 system (Millipore, USA). Hydrophilic interaction liquid chromatographic column (HILIC, 3.0 × 100 mm, 1.8 μm, Agilent Technologies Inc., CA, USA) was used for chromatographic separation. The column was maintained at 25°C and eluted at a flow rate of 0.5 ml/min. The mobile phase consisted of solvent A (25 mM ammonium acetate and 25 mM ammonium hydroxide in water) and solvent B (acetonitrile). The optimized gradient program was shown in Supplementary Table S1. The autosampler remained at 4°C. For mass data acquisition, the electrospray ionization (ESI) source of Triple TOF 6600 was operated in positive (ESI+) and negative ion (ESI–) modes. The main parameters of ESI source and mass spectrometry were used as follows: source temperature, 600°C; ion source gas1, 60 psi; ion source gas2, 60 psi; curtain gas, 30 psi; ion sapary voltage floating, ± 5,000 V (in both ESI modes), MS scan m/z range, 60–1,000 Da; MS scan accumulation time, 0.2 s per spectra. The MS/MS scan is acquired using information-dependent acquisition (IDA) with high sensitivity mode. The settings were as follows: product ion scan m/z range, 25–1,000 Da; product ion scan accumulation time, 0.05 s per spectra; collision energy, 35 ± 15 eV; declustering potential, ± 60 V (in both ESI modes); exclude isotopes within 4 Da, the maximum number of candidate ions to monitor per cycle, 10.

### Metabolomic Data Analysis

For metabolomics analysis, the raw mass spectrometry data were converted to common data format (.mzXML) using Proteowizard msConvert. Then, peak extraction and alignment were carried out by the XCMS program. Obtained data sets were normalized and Pareto-scaled, and imported into SIMCA-P V13.0 (Umetrics AB, Ume, Sweden) to perform unsupervised principal component analysis (PCA) and supervised partial least-squares discriminant analysis (PLS-DA) to screen and identify different metabolites. The quality and capability of PCA and PLS-DA models were described by cumulative R2 and Q2 (R2X: the fraction of the variables, R2Y: the goodness of the fit, Q2: prediction ability of the model). A permutation test with 200 permutations was performed to evaluate the robustness of the PLS-DA models. The variable importance in the projection (VIP) value from PLS-DA models was calculated to indicate the contribution of the variable to the classification. Potential metabolites with VIP > 1 were further applied to Student's *t*-test at the univariate level to measure its significance. Metabolites with VIP > 1 and *P* < 0.05 were selected and considered as potential biomarkers.

Accuracy m/z value of potential metabolites was searched against metabolite databases such as HMDB, METLIN, and Mass Bank (mass accuracy tolerance: 25 ppm). For the confirmation of the structure identification, the MS/MS spectra of the potential metabolites were compared with an in-house database established by available authentic standards. Metabolic pathway interpretation was conducted by MetaboAnalyst and Kyoto Encyclopedia of Genes and Genomes database.

### Deoxyribonucleic Acid Extraction and Sequencing

Total genome DNA from cecal content was extracted by using cetyltrimethylammonium bromide/sodium dodecyl sulfate (CATB/SDS) method, and the quality of DNA was monitored on 1% agarose gels. 16S rRNA genes were amplified using 341F/806R primer with the barcode (341F: 5′-CCTAYGGGRBGCASCAG-3′, 806R: 5′-GGACTACNNGGGTATCTAAT-3′). PCR reactions were performed in 30 μl reactions with 15 μl of Phusion® High-Fidelity PCR Master Mix (NEB, USA). The PCR conditions were 98°C for 1 min, followed by 30 cycles of 98°C for 10 s, 50°C for 30 s, 72°C for 60 s, and then 72°C for 5 min. PCR products were purified with AxyPrep DNA Gel Extraction Kit (Axygen, USA). According to the manufacturer's instruction, sequencing libraries were generated using NEB Next® Ultra™ DNA Library Prep Kit for Illumina (NEB, USA), and the index codes were added. With the application of Qubit@ 2.0 Fluorometer (Thermo Scientific, USA) and Agilent Bioanalyzer 2100 system (Agilent, USA), the quality of sequencing libraries was assessed. Finally, the library was sequenced on an Illumina HiSeq 2500 platform.

### Microbiomics Analysis

By using FLASH (http://ccb.jhu.edu/software/FLASH/), the raw paired-end reads from the original DNA fragments were merged. According to the unique barcodes, paired-end reads were assigned to each sample. The cluster of operational taxonomic units (OTUs) of the valid reads was performed by the UPARSE software package (19). Sequences with ≥97% similarity were assigned to the same OTUs. Based on the abundance of OTUs, the alpha diversity (Chao 1 and Ace) and beta diversity were analyzed with Perl scripts. Cluster analysis of cecal samples was preceded by weighted-Unifrac distance-based principal coordinate analysis (PCoA) using the QIIME software package (http://qiime.org/). Pearson's correlation test was used to assess the corrections between the liver metabolites and bacterial compositions, and the correlation heatmap was generated with ggplots package of R software.

### Statistical Analysis

Data are represented as mean ± SD. Comparisons of statistical significance among groups were made by one-way ANOVA with Fisher's least significant difference (LSD) test using the Statistical Package for Social Science program (SPSS 16.0, Chicago, IL, USA). A *p*-value < 0.05 was considered statistically significant.

## Results

### Identification of the Phytochemicals in the Decoction of ACD

**Figure 1:**
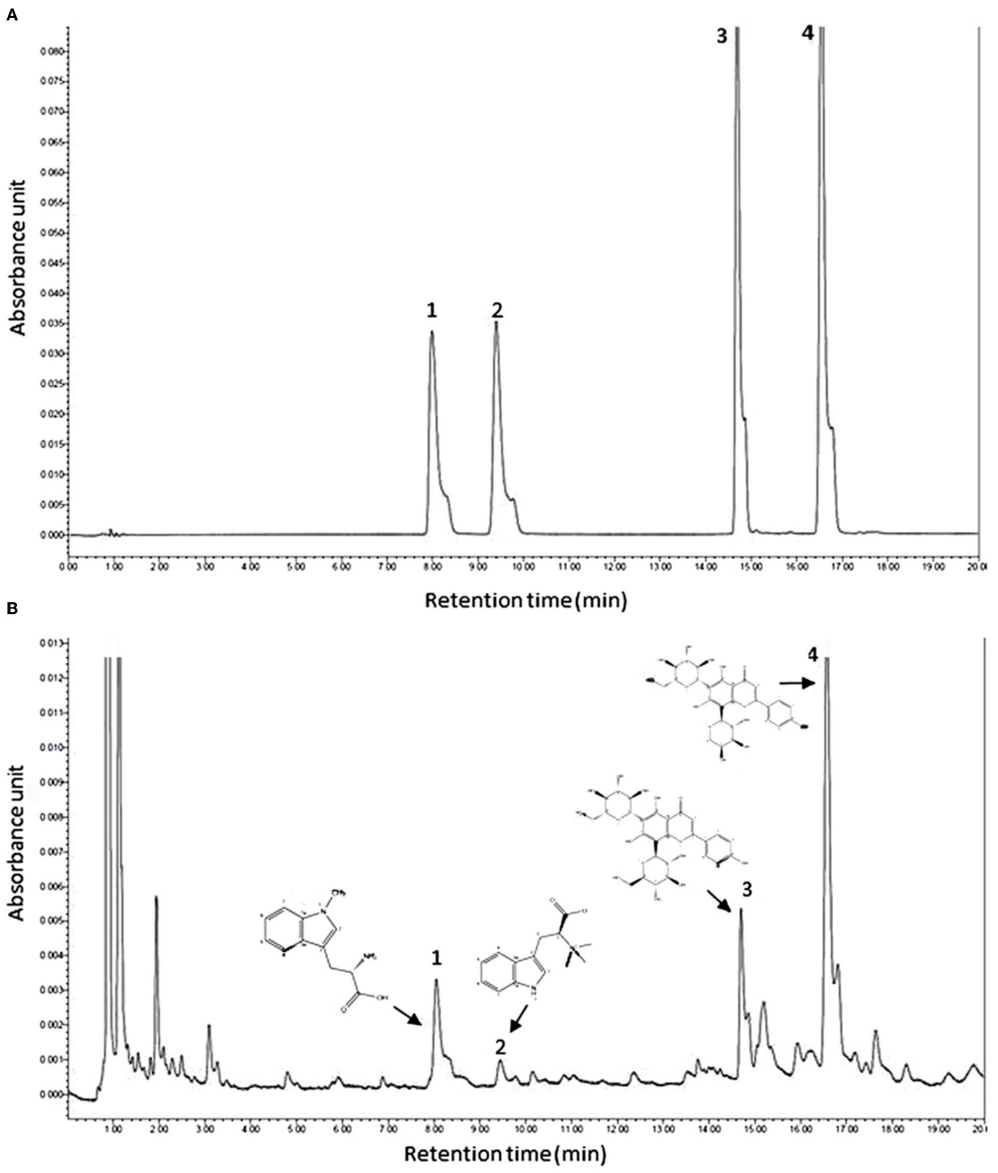
Typical ultra-high performance liquid chromatography chromatograms of mixed reference substances **(A)** and ACD **(B)**. ACD, *Abrus cantoniensis* Hance decoction; 1, abrine; 2, hypaphorine; 3, vicenin-2; 4, schaftoside.

The UPLC chromatogram of mixed standard solution and ACD are shown in Figure 1. Under the retention time of 20 min, chromatographic peaks of the active ingredient in ACD were clearly separated. Four phytochemicals, including abrine, hypaphorine, vicenin-2, and schaftoside, were identified, and the content of them in ACD was 3.49%, 0.81%, 6.13%, and 18.57%, respectively.

### Decoction of ACD Reduces Lipid Droplet Accumulation in the Liver

**Figure 2:**
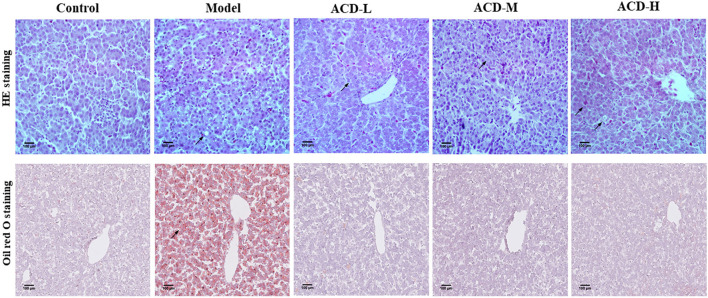
Effects of decoction of *Abrus cantoniensis* Hance (ACD) treatment on pathological results of the liver in laying hens. Liver sections were stained with HE and red oil O, the fatty degeneration and lipid droplet accumulation were indicated by the arrowhead. ACD treatment improved hepatic steatosis in laying hens with fatty liver hemorrhagic syndrome (FLHS).

Pathologic examination results of the liver stained by HE and oil red O are shown in Figure 2. In the control group, clear and normal liver cell architecture was observed. Serious fatty degeneration with lipid droplet accumulation and necrosis were found in laying hens with the HELP diet in the model group. In comparison with the model group, HELP diet-induced lipid accumulation in the liver from the laying hens with ACD treatment was decreased at different degrees, indicating the fatty degeneration was ameliorated by ACD. However, compared with the control group, the fat vacuoles, liver cell swelling, and cytoplasm rarefaction could be observed in ACD treatment groups (pointed by an arrow, Figure 2), suggesting the liver injury was not recovered back to normal state.

### Decoction of *Abrus cantoniensis* Hance Modulates Biochemical Blood Indicators

**Figure 3:**
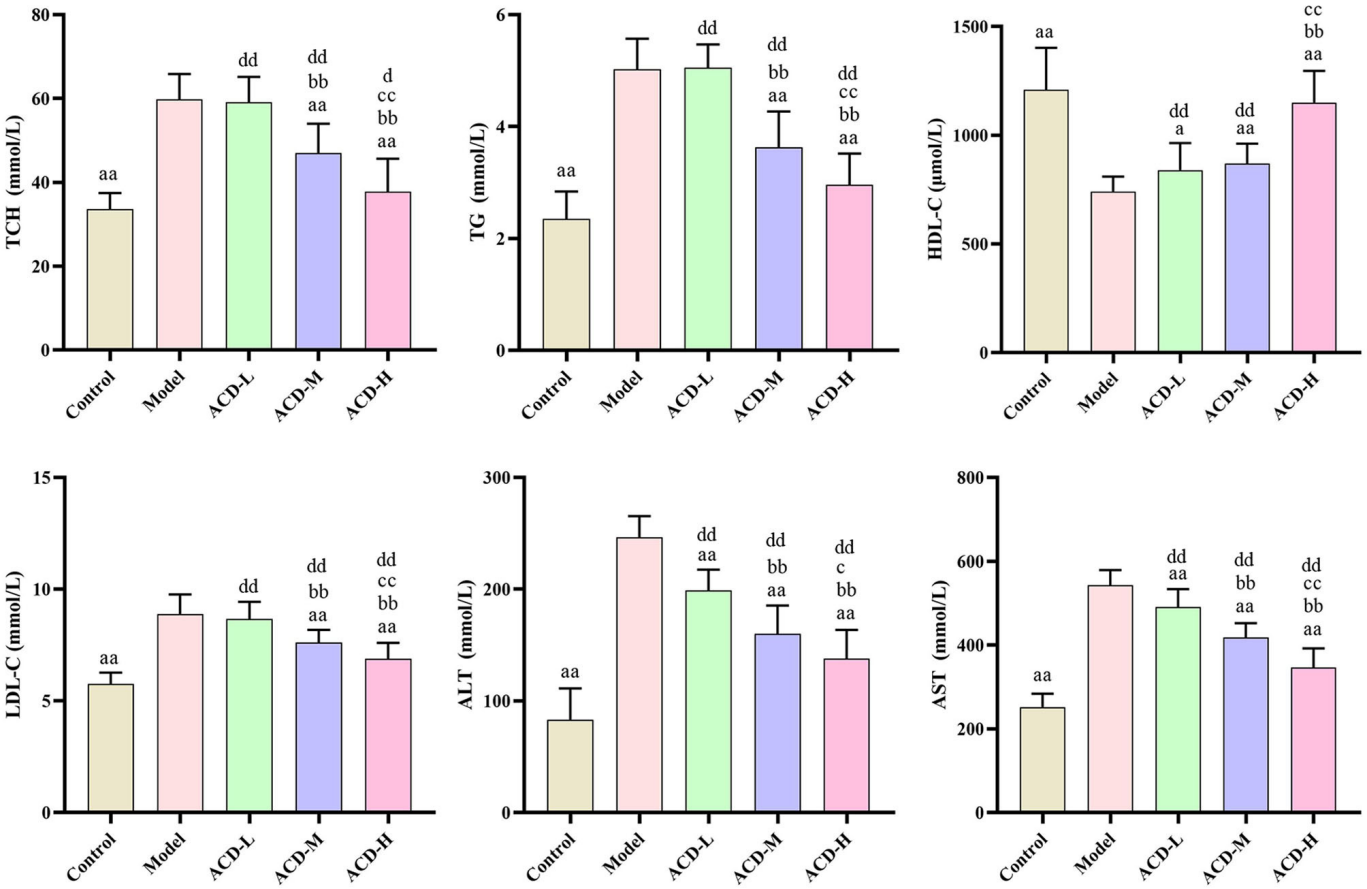
Effects of ACD on biochemical blood indicators in laying hens with FLHS (*n* = 8). TCH, total cholesterol; TG, triglycerides; HDL-C, high density lipoprotein cholesterol; LDL-C, low density lipoprotein cholesterol; ALT, alanine aminotransferase; AST, aspartate aminotransferase. ^a^*P* < 0.05, ^aa^*P* < 0.01, compared with model group; ^bb^*P* < 0.01, compared with ACD-L group; ^c^*P* < 0.05, ^cc^*P* < 0.01, compared with ACD-M group; ^d^*P* < 0.05, ^dd^*P* < 0.01, compared with control group.

As shown in Figure 3, compared with the control, AST, ALT, TG, LDL-C, and TCH increased significantly, while HDL-C decreased in laying hens fed with HELP diet in the model group (*P* < 0.01). Higher serum HDL-C and lower ALT and AST were detected in the ACD-L treatment group in comparison with the model (*P* < 0.01). Meanwhile, in ACD-M and ACD-H groups, it was found that the levels of AST, ALT, TG, LDL-C, and TCH were much lower and HDL-C were higher than those in the model (*P* < 0.01). In comparison to the control group, the levels of TCH, TG, LDL-C, AST, and ALT of laying hens in three ACD treatment groups were significantly higher (*P* < 0.01). HDL-C was markedly reduced in ACD-L and ACD-M groups than that in the control group (*P* < 0.01). However, no significant difference was observed for HDL-C between the control and ACD-H groups. The laying hens in ACD-M and ACD-H exhibited lower TG, TCH, ALT, LDL-C, and AST than those in the ACD-L group (*P* < 0.01). There was no difference in HDL-C between ACD-L and ACD-M groups. When compared with the ACD-M group, TCH, TG, LDL-C, ALT, and AST decreased significantly and HDL-C increased significantly in the ACD-H group, which showed that a high dose of ACD had a better reversing effect on biochemical blood indicators than low and medium dose ACD in laying hens fed with HELP diet. Due to the regulation effects of high dose ACD on biochemical indicators, the laying hens in the ACD-H group were used for further metabolomic analysis.

### Decoction of ACD Improves Liver Metabolic Profiling

To explore metabolic pathway changes related to ACD treatment, the metabolomics method was used to examine metabolite alterations in the liver. The datasets of the liver metabolomics study in both ESI+ and ESI- modes were provided in Supplementary File S1. Data quality is crucial for metabolomic study. In this study, the stability of the analytical method and instrument was monitored by the pooled QC samples. Results of PCA analysis on all liver samples are shown in Supplementary Figure S1. In PCA score plots, all of the QC samples were tightly clustered, which indicated the method and analytical system were robust with good stability and repeatability. Meanwhile, the separation among control, model, and ACD-H groups in both ESI+ and ESI- modes were evaluated by the unsupervised PCA. A slight but not significant separation trend among the laying hens in control, model, and ACD-H groups was observed in the PCA score plots (Supplementary Figure S1), suggesting the changes of the metabolomic profile of the liver from laying hens under different treatment.

**Figure 4:**
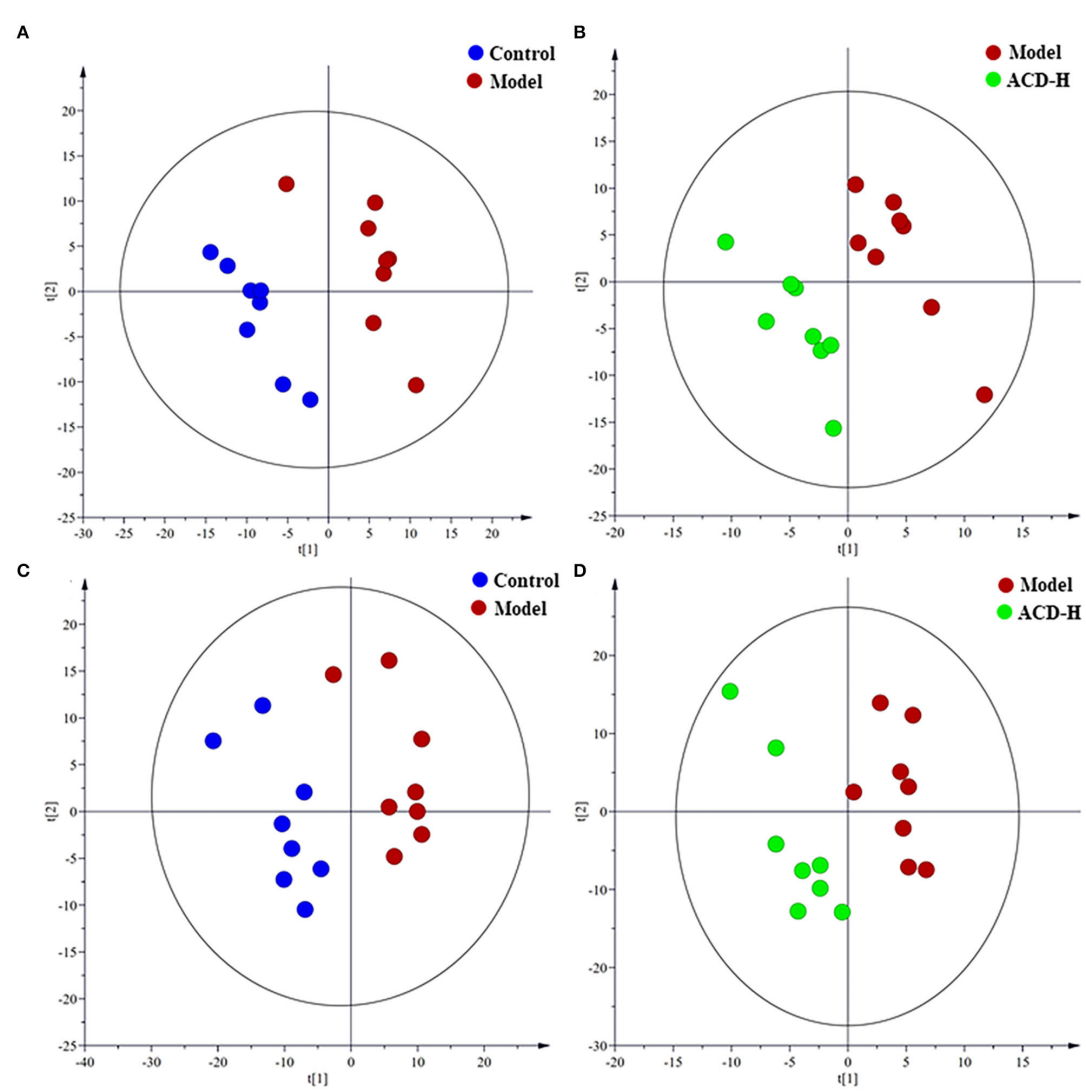
Partial least-squares discriminant analysis (PLS-DA) score plots of the laying hens in control, model and ACD-H groups (*n* = 8). **(A,B)** PLS-DA score plots in positive mode; Control vs. model: R^2^X = 0.32, R^2^Y = 0.90 and Q^2^ = 0.28; Model vs. ACD-H: R^2^X = 0.24, R^2^Y = 0.93 and Q^2^ = 0.22. **(C,D)** PLS-DA score plots in negative mode; Control vs. model: R^2^X = 0.38, R^2^Y = 0.88 and Q^2^ = 0.51; Model vs. ACD-H: R^2^X = 0.30, R^2^Y = 0.90 and Q^2^ = 0.15. Clear separation without overlap was observed among control, model and ACD-H groups.

Supervised PLS-DA was employed to identify the potential biomarker. The PLS-DA score plots are shown in Figure 4. A clear separation between control and model groups were observed in PLS-DA score plots in ESI+ and ESI- modes (Figures 4A,C), which indicated that significant metabolic disturbance occurred in the laying hens with FLHS. Meanwhile, the PLS-DA score plots showed that the liver samples in the ACD-H group were located far from those in the model (Figures 4B,D), revealing the metabolic disorders in the liver were improved by ACD treatment. The permutation test was performed to avoid the overfitting of the PLS-DA models. Validation plots of the PLS-DA permutation test showed that all the regression lines of Q2 (cum) points had a negative intercept (Supplementary Figure S2), which indicated the PLS-DA models were robust without overfitting.

### Metabolite Related With the Decoction of ACD Treatment and Pathway Analysis

**Figure 5:**
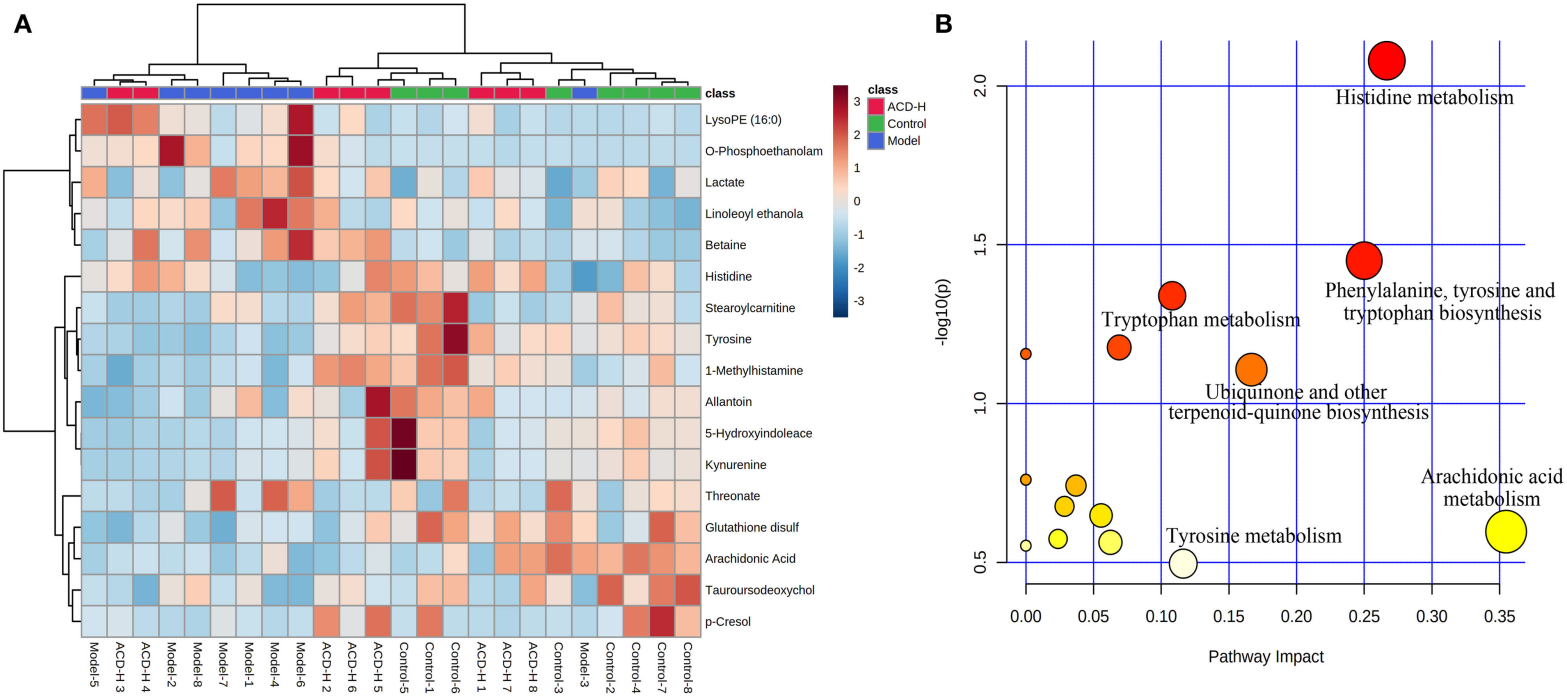
Heatmap and pathway analysis of the potential liver metabolites. **(A)** Hierarchical clustering analysis of differential liver metabolites. The relative abundances of the metabolites are represented by different colors: red indicates high abundance and blue indicates low abundance. **(B)** Disturbed pathways in response to ACD treatment. The larger bubble size represents higher pathway enrichment, and the darker color represents higher pathway-impact value.

Through the VIP and *P*-value analysis, 17 liver metabolites were identified as potential biomarkers associated with ACD treatment. Compound name, adduct, formula, fold change, and pathways of the metabolites are shown in Table 2. Compared with the control laying hens, the concentrations of 11 metabolites such as kynurenine, 5-hydroxyindoleacetate, arachidonic acid, histidine, tyrosine, and stearoylcarnitine were remarkably reduced, and 6 metabolites including lactate, LysoPE (16:0), betaine, and O-phosphoethanolamine were increased in the model group. Notably, ACD treatment reversed the abnormal liver metabolite changes in different degrees, such as the reduction of O-phosphoethanolamine and the increase of 1-methylhistamine and tyrosine. Hierarchical cluster analysis was carried out to fully and intuitively display the overview of the liver metabolites. The dendrogram in the vertical axis in Figure 5A showed that the liver samples were gathered into two clusters. Interestingly, six liver samples in the ACD-H group were clustered together with those in control, indicating the potential therapeutic effect of ACD on FLHS. As shown in the horizontal axis in Figure 5A, liver metabolites with similar abundance patterns were clustered together. For example, the metabolites such as LysoPE (16:0), O-phosphoethanolamine, lactate, linoleoyl ethanolamide, and betaine were clustered into one group.

**Table 2:** Potential biomarkers related to ACD treatment identified in the liver from laying hens with FLHS (*n* = 8).

**Metabolites**	**Adduct**	**Formula**	**m/z**	**RT (s)**	**Pathway**	**Fold change**	
						**M/C**	**ACD/M**
5-Hydroxyindoleacetate	(M-H)^−^	C_10_H_9_NO_3_	190.0504	257.31	Tryptophan metabolism	0.29^**^	1.62
Kynurenine	(M-H)^−^	C_10_H_12_N_2_O_3_	207.0767	257.01	Tryptophan metabolism	0.28^*^	1.81
Arachidonic Acid	(M-H)-	C_20_H_32_O_2_	303.2327	38.64	Arachidonic acid metabolism	0.63^*^	1.13
Lactate	(M-H)^−^	C_3_H_6_O_3_	89.0242	253.76	Gluconeogenesis	1.87^*^	0.75
p-Cresol	(M-H)^−^	C_7_H_8_O	107.0499	45.53		0.24^*^	2.58
Allantoin	(M-H_2_O-H)^−^	C_4_H_6_N_4_O_3_	139.0258	297.51		0.63^*^	1.31
Histidine	(M-H)^−^	C_6_H_9_N_3_O_2_	154.0619	413.48	Histidine metabolism	0.86	1.32^*^
Threonate	(M-H)^−^	C_4_H_8_O_5_	135.0295	327.41		1.02	0.52^*^
Tyrosine	(M+H)^+^	C_9_H_11_NO_3_	182.0783	251.83		0.33^**^	2.01^*^
1-Methylhistamine	(M+H)^+^	C_6_H_11_N_3_	126.1021	113.53		0.42^**^	2.12^*^
LysoPE (16:0)	(M+H)^+^	C_21_H_44_NO_7_P	454.2900	198.32	Phospholipid biosynthesis	2.65^*^	0.85
Glutathione disulfide	(M+H)^+^	C_20_H_32_N_6_O_12_S_2_	613.1573	490.07	Glutathione metabolism	0.62^*^	1.19
O-Phosphoethanolamine	(M+H)^+^	C_2_H_8_NO_4_P	142.0259	466.60		7.58^**^	0.35^*^
Stearoylcarnitine	(M-H+2Na)^+^	C_25_H_50_NO_4_	472.3387	166.18	Mitochondrial β-Oxidation of long chain saturated fatty acids	0.50^*^	1.14
Linoleoyl ethanolamide	(M+H)^+^	C_20_H_37_NO_2_	324.2884	36.20		1.70^**^	0.73
Tauroursodeoxycholic acid	(M+H-H_2_O)^+^	C_26_H_45_NO_6_S	482.2912	147.13	Bile acid metabolism	0.57^**^	1.11
Betaine	(M+H)^+^	C_5_H_12_NO_2_	118.0874	267.61		1.74^*^	0.98

*RT, retention time; LysoPE, lysophosphatidylethanolamine; M/C, model vs. control; ACD/M, ACD-H vs. model*.

* *P < 0.05*,

** *P < 0.01*.

Pathway analysis of the potential liver metabolites was performed to visualize the affected metabolic pathways (Figure 5B). The pathway with an impact value above 0.10 was selected out. The results revealed that arachidonic acid metabolism, histidine metabolism, phenylalanine, tyrosine and tryptophan biosynthesis, ubiquinone and other terpenoid-quinone biosyntheses, tyrosine metabolism, and tryptophan metabolism were associated with ACD treatment.

### Decoction of ACD Ameliorates Gut Microbiota Dysbiosis

**Figure 6:**
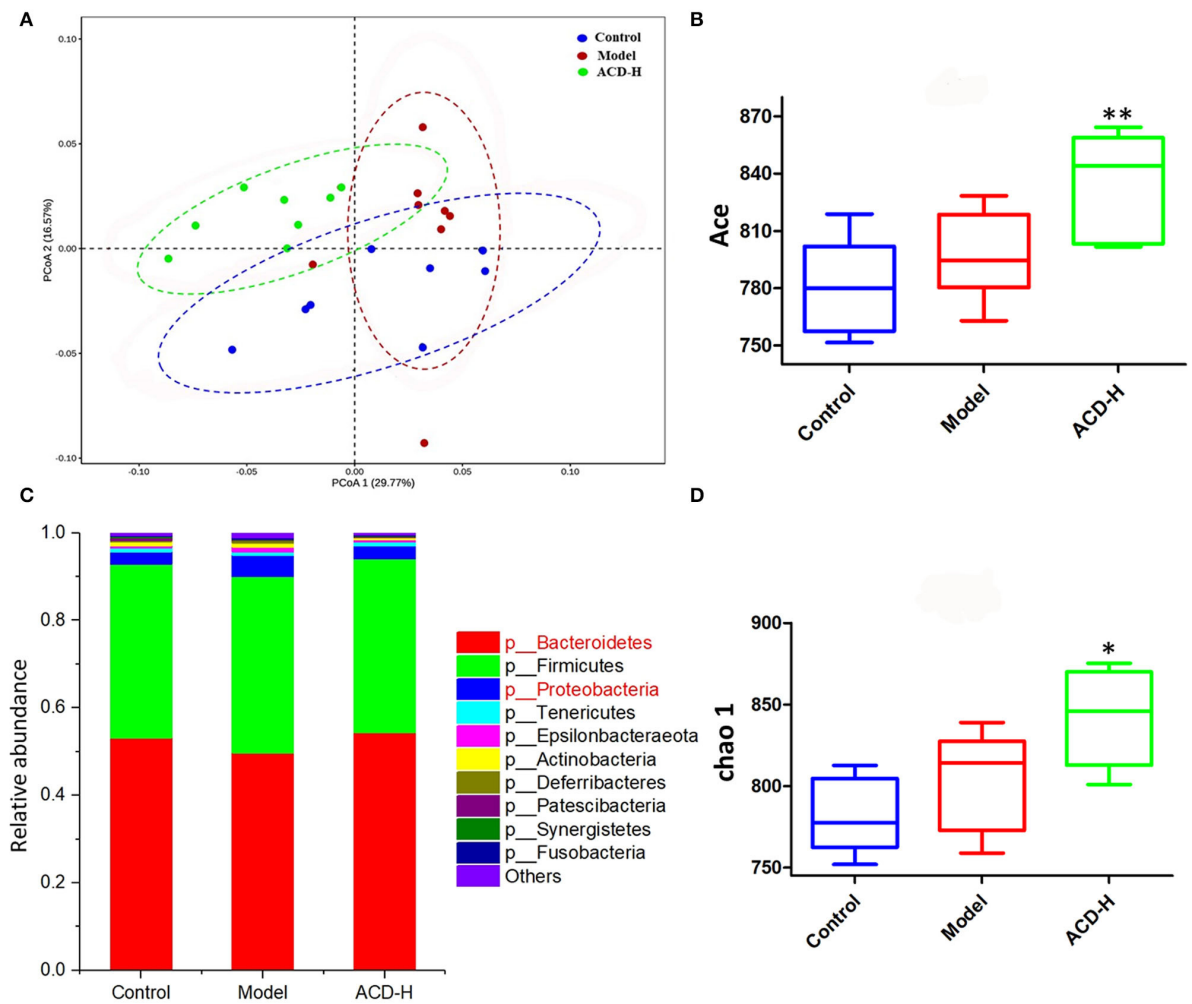
Effects of ACD on gut microbiota in laying hens with FLHS (*n* = 8). **(A)** Weighted-Unifrac distance-based principal coordinate analysis (PCoA) plots of bacterial community structures in the cecal content of laying hens from control, model and ACD-H groups. **(B)** ACE index in different groups. *P < 0.05, **P **<** 0.01, compared with model group. **(C)** Relative abundances of the gut microbiotas at the phylum level in cecal content in different groups. **(D)** Chao 1 index in different groups. ^*^P < 0.05, ^**^P < 0.01, compared with model group.

Gut microbiota dysbiosis is related to FLHS. In order to investigate the effects of ACD on gut microbiota composition, 16S rRNA gene sequencing was performed in this study. The summary of the sequencing data and rarefaction analysis are shown in Supplementary Table S2 and Supplementary Figure S3, respectively. These results indicated that high-quality sequencing data were obtained for further analysis. The relationship of the community structure of gut microbiota was investigated by the method of weighted-Unifrac distance-based PCoA, which revealed distinct clustering of rumen content samples in control, model, and ACD-H groups (Figure 6A). Notably, the samples in the ACD-H group were separated from the model, indicating ACD against the dysbiosis of microbiota community induced by HELP in laying hens. Next, Alpha diversities, including the Ace and Chao 1 index, were used to evaluate the changes in microbial communities (Figures 6B,D). Consistently, ACD treatment significantly increased Ace (*P* < 0.01) and Chao 1 (*P* < 0.05), which suggested ACD increased richness of the gut microbiota.

The relative abundance of gut bacterial composition at the phylum level is shown in Figure 6C. Compared with the control, the relative abundance of *Bacteroidetes* decreased, while *Proteobacteria* increased in the model group. Of note, the reduction of *Bacteroidetes* and the increase of *Proteobacteria* were inversed by ACD treatment at the phylum level (Figure 6C). At the genus level, according to the relative abundance and statistical difference, key microbial genera related to ACD treatment were selected (Table 3). When compared with the control group, abundance elevation of *Desulfovibrio, Erysipelatoclostridium*, and *Faecalibacterium* and decline of *Phascolarctobacterium* were observed in the laying hens fed with HELP in the model group (*P* < 0.01). Remarkable reduction of *Barnesiella, Desulfovibrio, Erysipelatoclostridium, Faecalibacterium, Negativibacillus*, and *Phascolarctobacterium* and increase of *Butyricicoccus, Prevotellaceae Ga6A1 group, Ruminococcaceae UCG-005*, and *Ruminococcaceae UCG-013* were found in the ACD-H group compared with those in the model (*P* < 0.01 and *P* < 0.05, Table 3).

**Table 3:** Relative abundance of rumen content microbiota at genus level (*n* = 8).

**Genus**	**Control**	**Model**	**ACD-H**
*Barnesiella*	0.34 ± 0.11	0.40 ± 0.17	0.23 ± 0.08^*^
*Butyricicoccus*	1.19 ± 0.19	1.22 ± 0.36	1.78 ± 0.41^**^
*Desulfovibrio*	2.06 ± 0.28^**^	4.17 ± 1.53	2.21 ± 0.62^**^
*Erysipelatoclostridium*	0.47 ± 0.16^**^	1.12 ± 0.64	0.56 ± 0.21^**^
*Faecalibacterium*	3.01 ± 0.56^*^	5.16 ± 3.29	2.22 ± 1.08^**^
*Negativibacillus*	0.35 ± 0.16	0.50 ± 0.22	0.18 ± 0.08^*^
*Phascolarctobacterium*	2.25 ± 0.60^**^	1.19 ± 0.53	0.62 ± 0.21^*^
*Prevotellaceae Ga6A1 group*	0.95 ± 0.56	0.86 ± 0.62	3.09 ± 2.56^**^
*Ruminococcaceae UCG-005*	2.44 ± 0.65	2.27 ± 0.57	4.66 ± 2.32^**^
*Ruminococcaceae UCG-013*	0.18 ± 0.05	0.22 ± 0.11	0.38 ± 0.08^**^

** *P < 0.01*,

* *P < 0.05 compared with model group*.

### Correlation Analysis of Metabolomics and Gut Microbiota

**Figure 7:**
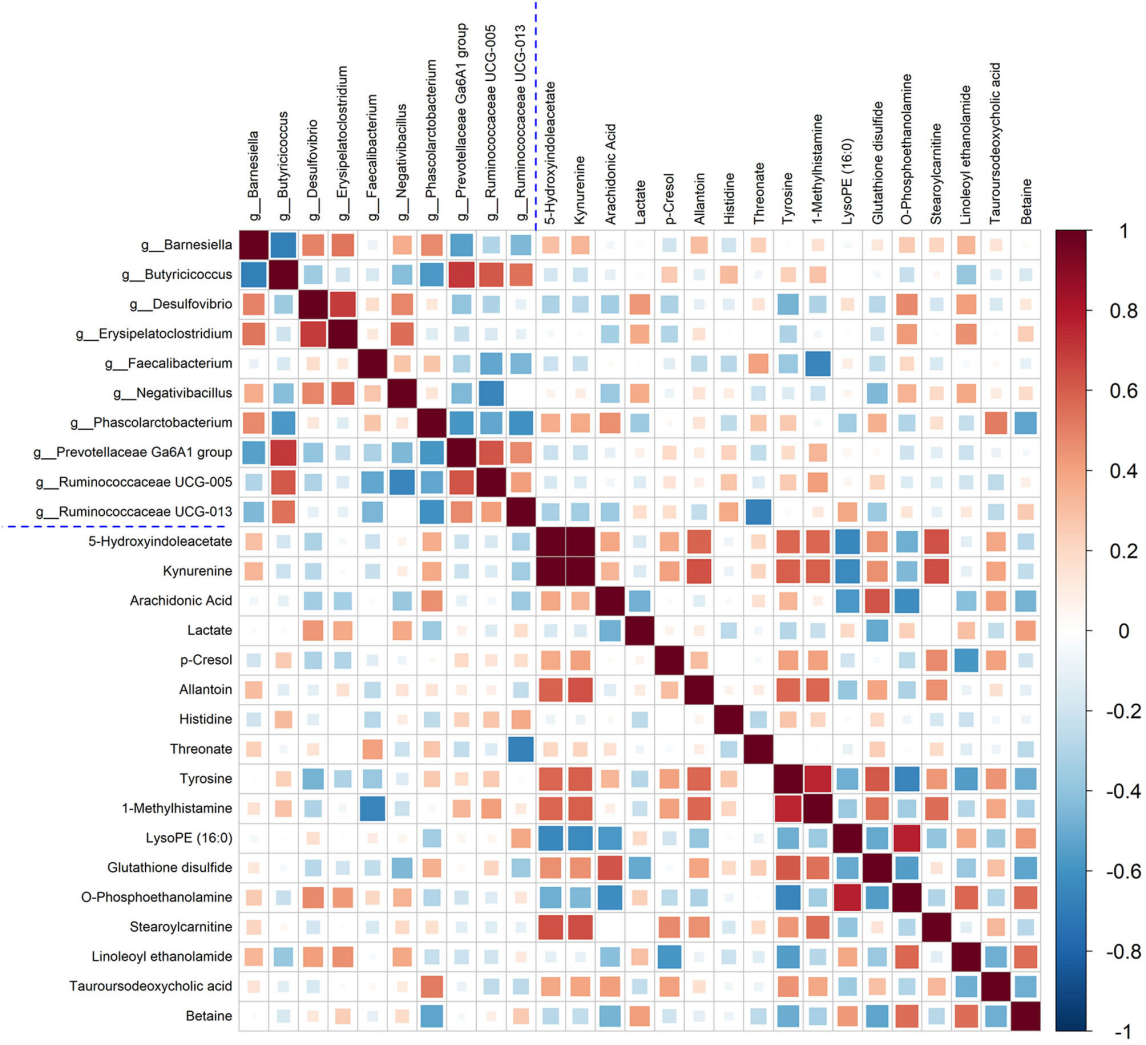
Correlation between the significantly changed liver metabolites and microbial genera. Pearson correlation coefficients between liver metabolites and microbiota genera were color-coded, that hot color indicating positive correlations, whereas the cold denoting negative correlations.

In this study, Spearman's correlation coefficients were calculated to investigate the correction between liver metabolites and gut microbiota. In Figure 7, the hot color (e.g., red) indicated the positive correlations between specific liver metabolites and certain bacterial genera, while the cool color (e.g., green) denoted the negative correlations. Interestingly, a clear correlation between liver metabolites and microbes in genus level in cecal content was observed. For example, Kynurenine and 5-Hydroxyindoleacetate showed positive corrections with *Barnesiella, Negativibacillus*, and *Phascolarctobacterium*. Relative abundance of genera *Faecalibacterium* and *Phascolarctobacterium* was positively correlated with arachidonic acid, threonate, and tauroursodeoxycholic acid. Histidine and tyrosine were positively correlated with *Butyricicoccus, Prevotellaceae Ga6A1 group*, and *Ruminococcaceae UCG-005*, but negatively with *Desulfovibrio* and *Faecalibacterium*.

## Discussion

Many studies indicated that the pathogenesis of FLHS is mainly related to inflammation activation, oxidative stress, and lipid metabolism alteration (4, 20). Phytochemicals in the ACD are the major bioactive compounds for therapeutic activity. In this study, four phytochemicals, including two alkaloids (abrine and hypaphorine) and two flavonoids (vicenin-2 and schaftoside) were identified in ACD. The pharmacologic activities such as antioxidation, anti-inflammation, hepatoprotective, and antihyperglycemic of the four phytochemicals have been proved in many studies (9, 21, 22). Targeted to the pathogeny of FLHS, it is easy to find that all the pharmacological activities of the components in ACD are beneficial for the treatment of FLHS. Additionally, the synergetic action among the phytochemicals in ACD may enhance the therapeutic effect. With the application of macroporous resin, Du et al. report that the content of vicenin-2 and schaftoside were 14.3% and 15.1% in the *Abrus mollis* extracts (23), which were higher than the result of vicenin-2 (6.31%) and lower than schaftoside (18.57%) in this study. The content of abrine (3.49%) in ACD was lower than the previous study in which abrine was quantified as 419.91 ± 0.4 mg/kg in methanol extract of AC (8). Different extraction methods, extract solvent, temperature, and time may be the reasons for the variation of phytochemical content in AC.

In laying hens, FLHS induced by feeding HELP is a classic disease model (24). Rozenboim et al. observed that young hens aged 15 weeks were more susceptible to a low-protein high-fat diet than the hens aged 80 weeks, which were confirmed by higher liver color score, liver hemorrhagic score, and liver fat content in young hens (25). Therefore, hens aged 90 days were used for the FLHS disease model in this study. Disorder of blood lipids and hepatic steatosis are the main features of FLHS, which can lead to the elevation of TG, TCH, and LDL-C, reduction of HDL-C, and lipid accumulation in the liver (26). ALT and AST are important liver function biochemical indicators, which can reflect the degree of liver damage. As lipoprotein, HDL-C and LDL-C are playing important roles in the uptake and transport of lipids and cholesterol in the bloodstream, liver, and tissue (27). It has been proved that the improvement of blood lipid levels, such as the reduction of TG, TCH, and HDL-C and the increase of HDL-C, contributes to reducing liver lipid accumulation and damage in laying hens with FLHS (28). In this study, abnormal blood lipid levels and typical pathologic changes of the liver were observed in the laying hens fed with the HELP diet, suggesting the FLHS model was successfully established. Interestingly, from the results of biochemical and pathologic analysis in this study, ACD treatment ameliorated the blood lipid disorder and pathological liver changes in laying hens fed with HELP diet. Moreover, it was also noted that ACD regulated the levels of ALT, AST TG, TCH, LDL-C, and HDL-C in a dose-dependent manner. Additionally, in ACD treatment groups, results of blood biochemistry and pathological liver changes were agreed with each other, indicating the ameliorative effects of ACD on FLHS.

As a sensitive and powerful tool, metabolomics is widely used to reveal disease pathogenesis and the action mechanism of herb medicine (29, 30). Previous metabolomics studies found that the metabolism of lipid, amino acid, and glucose was involved in the progression of FLHS in laying hens (14). In this study, the liver metabolomics results found that there was a significant difference in the metabolomics patterns in PCA and PLS-DA score plots among control, model, and ACD-H groups, which was partly agreed with the results of blood lipid and liver pathologies. Moreover, seventeen different abundance metabolites (e.g., arachidonic acid, LysoPE (16:0), histidine, tyrosine, and kynurenine) that involved in arachidonic acid, lipid, and amino acid metabolism were identified and related with ACD treatment. Arachidonic acid, an essential fatty acid, is the precursor of numerous important biological mediators such as prostaglandins, thromboxanes, and leukotrienes. Several publications have demonstrated that arachidonic acid level was downregulated in rats with nonalcoholic fatty liver disease (NAFLD) and patients with cardiovascular disease (31, 32). In this study, arachidonic acid was reduced in the liver from the laying hens with FLHS, which was matched with the above-mentioned reports. A favorable increase of arachidonic acid was observed in ACD treated laying hens, indicating that the regulation effect of ACD on arachidonic acid metabolism might contribute to its efficacy on FLHS. LysoPEs are closely associated with inflammation, hyperlipidemia, atherosclerosis, and fatty liver diseases (33, 34). In this study, compared with the control, LysoPE (16:0) was significantly increased in the model group, suggesting the disorder of lipid metabolism in laying hens with FLHS. Increased LysoPE (16:0) was inhibited by ACD-H treatment, implying that the therapeutic effect of ACD on FLHS might ascribe to the inhibition of lipid metabolism. A lower level of tyrosine was observed in the model group than that in control, which was consistent with the previous reports that tyrosine was reduced in the liver in the mice fed with a high-fat diet (35). Song et al. reported that histidine supplementation could relieve liver injury induced by a high-fat diet in rats, and the increased intake of histidine might be a potential therapeutic choice for NAFLD (36). In this study, reduced levels of tyrosine and histidine in liver tissue in the model group might be caused by the lack of protein in the HELP diet. In contrast to the model, tyrosine and histidine were significantly increased after ACD-H treatment, which implied that ACD could improve amino acid against FLHS in laying hens.

A large number of previous findings indicated that gut microbiota dysfunction is a strong link to liver disease and may be a potential therapeutic target for liver disease treatment (37). Our PCoA score plots showed that the samples in the ACD-H group were located away from those in the model, suggesting ACD treatment altered the community structure of cecal microbiota. Chao 1 and Ace are estimators of species richness. Remarkably, increased Chao 1 and Ace indicated that ACD treatment increased the richness of gut microbiota, which contributes to the improvement of the gut microbial community. At the phylum level, a substantial decrease in *Bacteroidetes* and a rise in *Proteobacteria* were observed in the model group in this study. The finding of *Bacteroidetes* agrees with previously published literature that the relative abundance of *Bacteroidetes* was significantly lower in fatty liver disease (38). Shin et al. reported that the increased percentage of *Proteobacteria* could reflect the dysbiosis of gut microbiota and the instability of the gut microbial community (39). Compared to the laying hens with FLHS, ACD modified the relative abundance of *Bacteroidetes* and *Proteobacteria*, indicating the modulation effects of ACD on gut microbiota.

A previous study proved that the relative abundance of *Butyricicoccus* was significantly reduced in mice fed with a high-fat diet and negatively correlated with serum lipid levels (40). So, increased *Butyricicoccus* in the ACD-H group was beneficial for reducing blood lipids. Evidence demonstrated that *Desulfovibrio* was strongly correlated with obesity, metabolic syndrome, and inflammation, and increased *Desulfovibrio* was reported in C57BL/6 mice with NAFLD (41), supporting the changes of *Desulfovibrio* in this study. Conversely, in ACD treated laying hens, the relative abundance of *Desulfovibrio* was lower than that in the model, which might have beneficial effects on FLHS treatment. *Erysipelatoclostridium*, a bad Gram-positive bacillus, can produce IgA1 and IgA2 proteases to increase the risk of invasive infections caused by opportunistic bacterial invasion (42). A significant reduction of *Erysipelatoclostridium* was observed in the ACD-H group, which might reduce infection risk to keep the host healthy. In addition, through the correlation analysis, it was found that there was a possible link between the altered gut microbiota and liver metabolites in laying hens with ACD treatment. However, the sophisticated interaction mechanisms among ACD, liver metabolites, and gut bacteria deserve further study.

## Conclusion

In conclusion, ACD had protective effects on FLHS in laying hens induced by the HELP diet through regulating blood lipids, reducing liver lipid accumulation, and ameliorating the dysbiosis of liver metabolomics and gut microbiota. This study was beneficial to understanding the effects and action mechanism of AC and provided new evidence for the application of AC in FLHS treatment.

## Data Availability Statement

The datasets presented in this study can be found in online repositories. The names of the repository/repositories and accession number(s) can be found at: National Center for Biotechnology Information (NCBI) BioProject database under accession number PRJNA801433.

## Ethics Statement

The animal study was reviewed and approved by Institutional Animal Care and Use Committee of Hebei Agricultural University.

## Author Contributions

XL and YP performed the experiments and analyzed the data. HL and XZ supplied reagents. NM and JL designed the experiments. XL and NM wrote the manuscript. YS provided the writing guidance and revised the manuscript. All the authors have read and approved the final version of the manuscript.

## Funding

This study was supported by the Science-Technology Innovation Engineering of CAAS (25-LZIHPS-02), Natural Science Foundation of Hebei Province (C2021204035), and the Hebei Layer and Broiler Innovation Team of Modern Agro-industry Technology Research System (HBCT2018150210).

## Conflict of Interest

The authors declare that the research was conducted in the absence of any commercial or financial relationships that could be construed as a potential conflict of interest.

## Publisher's Note

All claims expressed in this article are solely those of the authors and do not necessarily represent those of their affiliated organizations, or those of the publisher, the editors and the reviewers. Any product that may be evaluated in this article, or claim that may be made by its manufacturer, is not guaranteed or endorsed by the publisher.

## Supplementary Material

Supplementary Table S1 Optimized gradient elution program of ultra-high performance liquid chromatography (UPLC)-Q-TOF/MS in liver metabolomic analysis.

Supplementary Table S2 Summary of the sequencing data of cecal content.

Supplementary File S1 The metabolomics datasets of liver samples in positive and negative modes by ultra-high performance liquid chromatography (UPLC)-Q-TOF/MS analysis.

Supplementary Figure S1 Principal component analysis (PCA) score plots of electrospray ionization (ESI) positive and negative modes based on ultra-high performance liquid chromatography (UPLC)-Q-TOF/MS analysis of liver samples.

Supplementary Figure S2 Permutation test of the corresponding partial least-squares discriminant analysis (PLS-DA) models.

Supplementary Figure S3 Rarefaction curve of the cecal content samples.
