# Supplementary material for: Protective Effects of Abrus cantoniensis Hance on the Fatty Liver Hemorrhagic Syndrome in Laying Hens Based on Liver Metabolomics and Gut Microbiota
Source: Front Vet Sci. 2022 Apr 15;9:862006. doi: 10.3389/fvets.2022.862006 (PMC9051509; doi:10.3389/fvets.2022.862006)

Table S1 Optimized gradient elution program of UPLC-Q-TOF/MS in liver metabolomic analysis.

| Time (min) | Solvent A (%) | Solvent B (%) |
| --- | --- | --- |
| 0 | 5 | 95 |
| 0.5 | 5 | 95 |
| 7 | 35 | 65 |
| 8 | 60 | 40 |
| 9 | 60 | 40 |
| 9.1 | 5 | 95 |
| 12 | 5 | 95 |

Solvent A: 25 mM ammonium acetate and 25 mM ammonium hydroxide in water; solvent B: acetonitrile.

Figure S1 PCA score plots of ESI positive and negative modes based on UPLC-Q-TOF/MS analysis of liver samples.
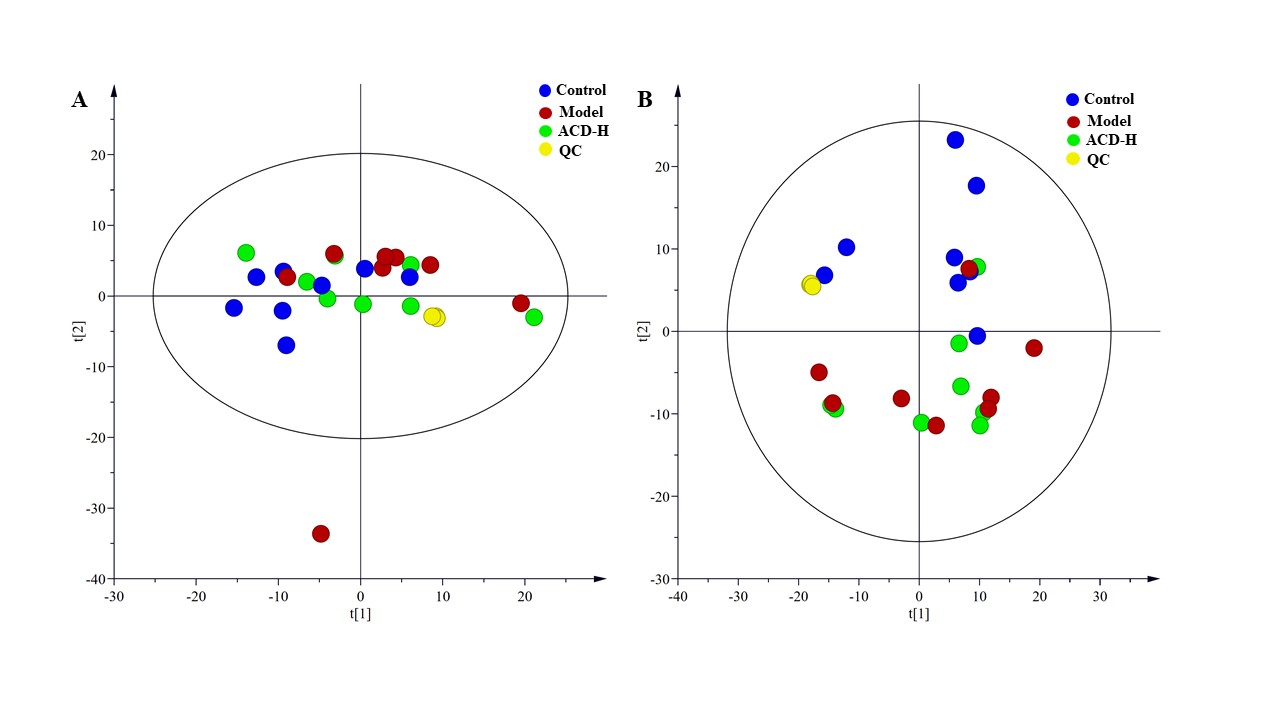


PCA: principal component analysis; A: ESI positive mode; B: ESI negative mode. All the QC samples (Yellow) were tightly clustered, indicating the method and analytical system used for metabolomic study were robust with good stability and repeatability.

Figure S2 Permutation test of the corresponding PLS-DA models.


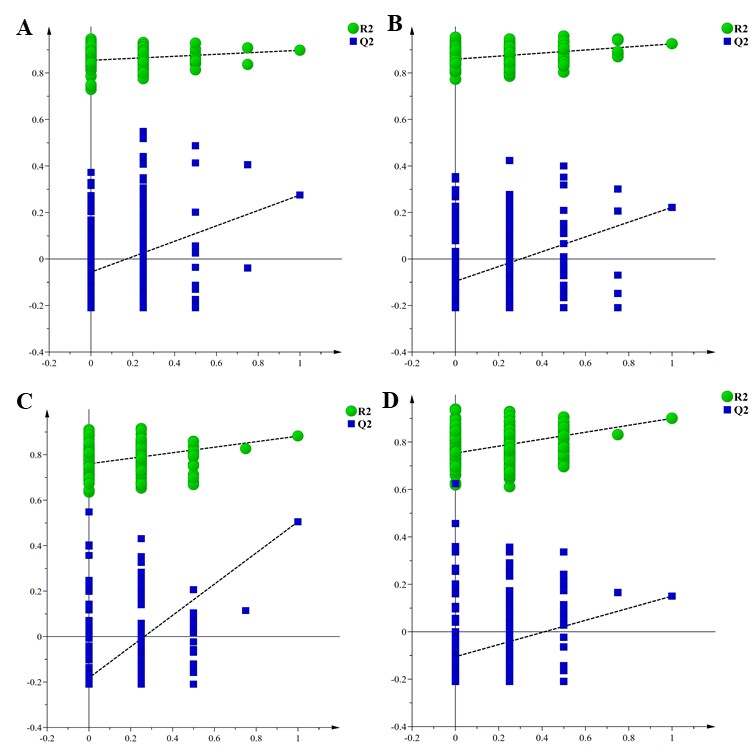


**Model versus ACD-H**

**Control versus model**

**ESI+**

**ESI-**

A 200-times permutation test of the corresponding PLS-DA models. A and B: permutation test in positive mode (ESI+). A, control versus model, the Y-axis intercepts were: R2 (0, 0.86), Q2 (0, -0.06); B, model versus ACD-H, the Y-axis intercepts were: R2 (0, 0.86), Q2 (0, -0.10); C and D: permutation test in negative mode (ESI-). C, control versus model, the Y-axis intercepts were: R2 (0, 0.76), Q2 (0, -0.18); D, model versus ACD-H, the Y-axis intercepts were: R2 (0, 0.75), Q2 (0, -0.10).

| Sample ID | Raw PE reads | Raw Tags | Clean Tags | Effective Tags | Q20 | Q30 | GC% |
| --- | --- | --- | --- | --- | --- | --- | --- |
| Control-1 | 82,627 | 75,854 | 74,599 | 66,113 | 98.17 | 94.15 | 53.5 |
| Control-2 | 94,803 | 85,962 | 84,614 | 74,255 | 98.42 | 94.87 | 53.01 |
| Control-3 | 99,615 | 93,159 | 91,737 | 79,951 | 98.29 | 94.56 | 53.2 |
| Control-4 | 89,241 | 82,578 | 81,382 | 71,343 | 98.36 | 94.73 | 53.06 |
| Control-5 | 93,382 | 86,460 | 85,167 | 74,907 | 98.31 | 94.49 | 53.46 |
| Control-6 | 97,307 | 91,133 | 89,781 | 79,176 | 98.3 | 94.5 | 53.18 |
| Control-7 | 82,279 | 75,872 | 74,694 | 64,645 | 98.23 | 94.31 | 52.43 |
| Control-8 | 81,730 | 75,931 | 74,927 | 62,433 | 98.48 | 94.96 | 52.51 |
| Model-1 | 88,802 | 81,523 | 80,162 | 70,816 | 98.17 | 94.21 | 53.09 |
| Model-2 | 84,764 | 77,623 | 76,332 | 68,118 | 98.23 | 94.28 | 53.63 |
| Model-3 | 91,628 | 85,457 | 84,065 | 75,823 | 98.43 | 94.94 | 54.12 |
| Model-4 | 86,838 | 80,293 | 79,014 | 69,871 | 98.29 | 94.56 | 53.85 |
| Model-5 | 68,103 | 61,760 | 60,766 | 52,995 | 98.56 | 95.25 | 52.85 |
| Model-6 | 99,050 | 89,725 | 88,206 | 79,439 | 98.05 | 93.8 | 53.81 |
| Model-7 | 93,606 | 86,809 | 85,533 | 76,275 | 98.31 | 94.57 | 52.72 |
| Model-8 | 81,169 | 75,131 | 74,135 | 63,858 | 98.41 | 94.74 | 52.58 |
| ACD-H1 | 92,724 | 85,412 | 84,033 | 74,629 | 98.25 | 94.44 | 53.02 |
| ACD-H2 | 87,006 | 79,381 | 78,218 | 69,473 | 98.49 | 95.09 | 53.04 |
| ACD-H 3 | 98,086 | 89,657 | 88,024 | 76,974 | 98.43 | 94.95 | 52.88 |
| ACD-H4 | 93,409 | 86,389 | 85,062 | 75,368 | 98.38 | 94.79 | 53.47 |
| ACD-H5 | 95,577 | 88,181 | 86,841 | 77,997 | 98.25 | 94.33 | 53.18 |
| ACD-H6 | 82,289 | 74,968 | 73,605 | 66,251 | 98.01 | 93.8 | 52.83 |
| ACD-H7 | 83,540 | 76,080 | 74,758 | 67,143 | 98.02 | 93.73 | 52.28 |
| ACD-H8 | 94,616 | 88,516 | 87,211 | 75,736 | 98.55 | 95.21 | 51.76 |

Table S2 Summary of the sequencing data of cecal content.

Figure S3 Rarefaction curve of the cecal content samples.

Observed species gradually increased with the increase of sequence number, and the curve became gentle, indicating the obtained sequence data could reflect the abundance and diversity of the microbiota.
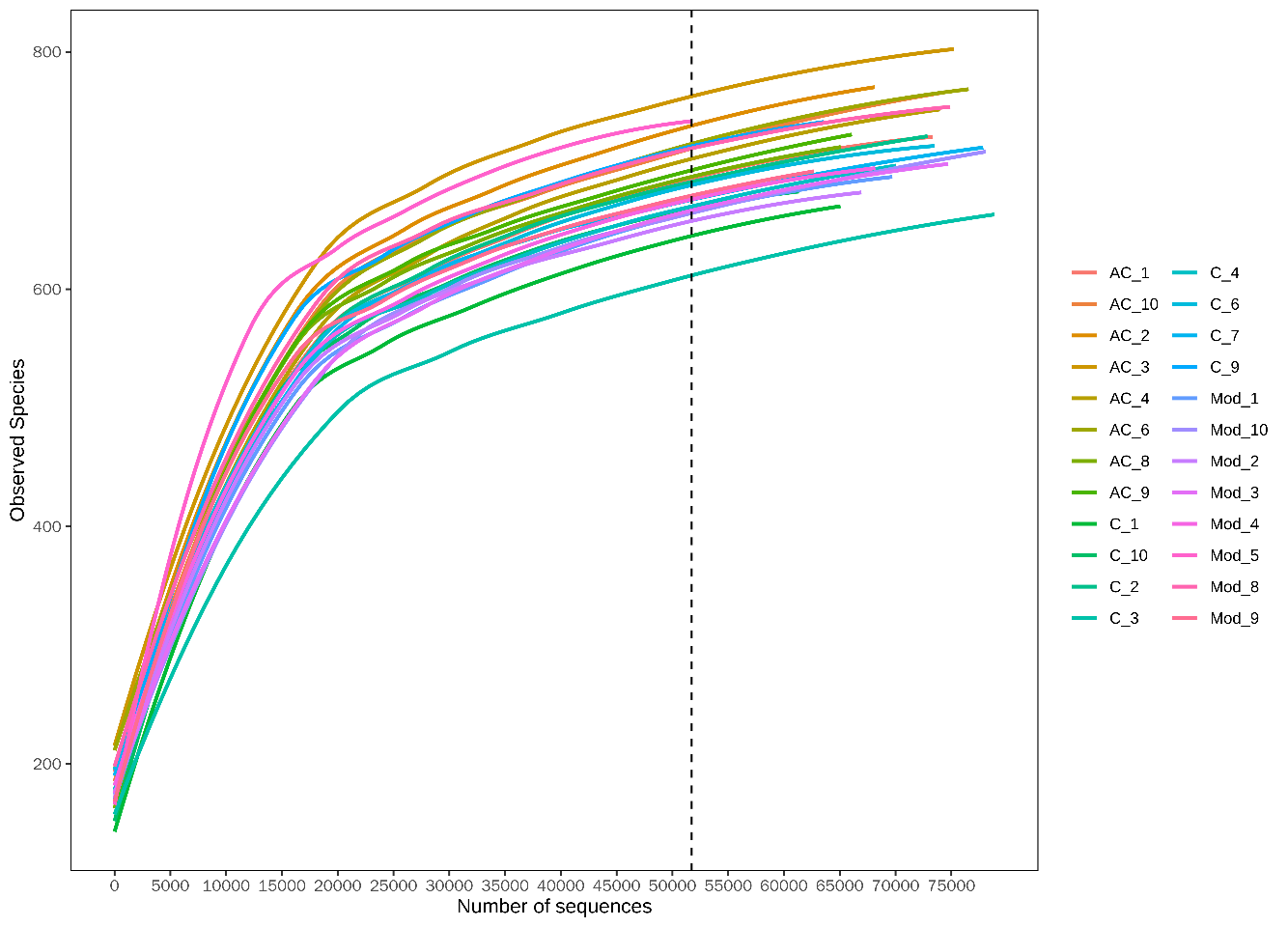

Supplement: Supplementary Table S2 — Summary of the sequencing data of cecal content. [file Table_2.DOCX]
